# Clock drawing test: comparison between the Pfizer and the Shulman systems

**DOI:** 10.1590/1980-57642021dn15-040009

**Published:** 2021

**Authors:** Daniela Bertol Graeff, Jéssica Maldaner Lui, Nathália Dal Prá Zucco, Ana Luisa Sant’Anna Alves, Cassiano Mateus Forcelini, Bernadete Maria Dalmolin

**Affiliations:** 1 Universidade de Passo Fundo – Passo Fundo, RS, Brazil.

**Keywords:** cognitive decline, screening, clock drawing test, correlation, declínio cognitivo, programas de rastreamento, teste do desenho do relógio, correlação

## Abstract

Cognitive decline can be screened by the clock drawing test (CDT), which has several versions.

**Objective:**

This survey aimed to analyze the correlation between two simple methods for scoring the CDT.

**Methods:**

This cross-sectional study was nested in the Elo-Creati cohort from Passo Fundo, Brazil and comprised 404 subjects. Two raters underwent previous training and scored the subjects’ CDT according to both the Pfizer and Shulman systems. The inter-observer and intra-observer concordance within each method was analyzed with the Spearman’s rank correlation coefficient, as well as the concordance of the scores between the two methods. Age and scholarity were also correlated with the scores.

**Results:**

Most of the participants were women (93.8%) and Caucasian (84.6%), with a mean age of 66.9 (±7.8) years and a scholarity of 10.9 years (±5.6). There was significant inter-observer (Pfizer: r=0.739, p£0.001; Shulman: r=0.727, p£0.001) and intra-observer correlation (Pfizer: rater 1, r=0.628, p≤0.001; rater 2, r=0.821, p≤0.001; Shulman: rater 1, r=0.843, p≤0.001; rater 2: r=0.819; p≤0.001). Intra-observer correlation was also observed comparing Pfizer and Shulman methods (rater 1: r=0.744; p≤0.001; rater 2: r=0.702; p≤0.001). There was weak correlation of the scores with scholarity (Pfizer: r=0.283, p£0.001; Shulman: r=0.244, p£0.001) and age (Pfizer: r=-0.174, p£0.001; Shulman: r=-0.170, p£0.001). More participants were classified with decreased cognition through the Pfizer system (rater 1: 44.3 vs. 26.5%; rater 2: 42.1 vs. 16.3%; p≤0.001).

**Conclusions:**

For this population, our results suggest that the Pfizer system of scoring CDT is more suitable for screening cognitive decline.

## RESUMO

O déficit cognitivo pode ser triado pelo teste do desenho do relógio (TDR), que tem várias versões.

**Objetivo:**

Esta pesquisa visou avaliar a concordância entre dois métodos simples de TDR.

**Métodos:**

Estudo transversal, aninhado na coorte Elo-Creati de Passo Fundo, Brasil, que incluiu 404 sujeitos. Dois avaliadores previamente treinados analisaram o TDR dos participantes de acordo com os sistemas de Pfizer e de Shulman. A concordância inter e intraobservador foi analisada com o teste de coeficiente de correlação de postos de Spearman, assim como a concordância pela estatística kappa dos escores entre os métodos. Idade e escolaridade também foram correlacionados com os escores.

**Resultados:**

A maioria dos participantes era de mulheres (93,8%) e caucasianos (84,6%), com média de idade de 66,9±7,8 anos e de escolaridade de 10,9±5,6 anos. Houve significativa correlação interobservador (Pfizer: r=0,739, p£0,001; Shulman: r=0,727, p£0,001) e intraobservador (Pfizer: avaliador 1, r=0,628, p≤0,001; avaliador 2, r=0,821, p≤0,001; Shulman: avaliador 1, r=0,843, p≤0,001; avaliador 2: r=0,819; p≤0,001). Correlação intraobservador significativa também foi evidenciada comparando-se os sistemas de Pfizer e Shulman (avaliador 1: r=0,744; p≤0,001; avaliador 2: r=0,702; p≤0,001). Houve fraca correlação dos escores com escolaridade (Pfizer: r=0,283, p£0,001; Shulman: r=0,244, p£0,001) e idade (Pfizer: r=-0,174, p£0,001; Shulman: r=-0,170, p£0,001). Mais participantes foram classificados com declínio cognitivo com o sistema de Pfizer (avaliador 1: 44,3 *vs*. 26,5%; avaliador 2: 42,1 *vs*. 16,3%; p≤0,001).

**Conclusões:**

Nossos resultados sugerem que, para essa população, o sistema de Pfizer para avaliar o TDR é mais adequado para a triagem cognitiva.

## INTRODUCTION

The number of older people, including those living with cognitive decline and dementia, is rising.^1^ The majority of older adults with dementia live in low- and middle-income countries, and such a preponderance will increase over the next decades.^2^ Early identification of cognitive decline is desirable to allow adequate management and improve outcomes.^3^

There are several cognitive screening tools for identifying cognitive decline and dementia, but the validation of these tests in illiterate and low-educated older adults is a challenge.^4,5^ One of the most simple of them is the clock drawing test (CDT), which has more than one version with different scoring methods. The 5-item score Shulman system was considered as an accurate method for the widespread use in the diagnosis of dementia, requiring a substantial understanding of its scoring system.^6^ In contrast, small cross-sectional studies suggested that CDT is not sensitive enough to detect very mild dementia neither in educated people^7^ nor in those with limited education,^5^ even though the CDT version employed in the last study had a complicated scoring system. Moreover, additional uses for the several CDT versions have been described in terms of evaluating the progression of cognitive decline,^8^ differentiating types of dementia,^9^ and even classifying qualitatively the elements of CDT according to the educational level.^10^

The issue about the usefulness of the CDT is far from being clarified. In this setting, this survey aimed to analyze the correlation between two simple methods for scoring the CDT in a large series of adult and elderly people, as well as their inter- and intra-observer correlations.

## METHODS

This cross-sectional study was nested in the Elo-Creati (*Estudo Longitudinal do Centro de Referência e Atenção ao Idoso*) cohort from the urban area of Passo Fundo, RS, Brazil. This study has been accomplished by the Universidade de Passo Fundo (UPF) and comprises 404 adult and elderly subjects from the community who have been followed since 2014. The Elo-Creati cohort has the objective of accompanying their health status and promoting well-being through the intervention of professors and students from areas related to health care with experience in the application of cognitive tests. All subjects were invited to participate in this study, gave their written consent, and were evaluated between 2014 and 2015. No patient subject declined participation during the period of this research, which was conducted in accordance with the Declaration of Helsinki and was approved by the Ethical Committee of the UPF (Report Number 741.214).

Demographic and clinical data were obtained from a scheduled interview with each subject performed in a quiet and comfortable room, followed by the task of CDT with a pencil on a white paper, with the clock indicating “10 min to 2 h.” Only the final drawing was evaluated, with no concern about the speed and agility for performing it, according to the classical Pfizer and Shulman systems. For both, the higher numbers indicate better performance. The Pfizer system consists of a 4-item scale evaluation, where one point is assigned for drawing a closed circle, one point for including all 12 numbers, one point for placing the numbers in correct positions, and finally the last point for disposing of the clock-hands adequately.^7^ The Shulman system implies five points for a perfect clock, four points for minor visuospatial errors, three points for inaccurate representation of 10 min to 2 h when the visuospatial organization is well done, two points for moderate visuospatial disorganization of numbers such that accurate denotation of 10 min to 2 h is impossible, one point for a severe level of visuospatial disorganization, and zero point for inability to make any reasonable representation of a clock.^11^ The cut-off score for considering a drawing as abnormal according to the Pfizer method was any score different from 4,^12^ while the corresponding score in the Shulman system was any score below 4.^13^

Two medicine students underwent previous training performed by an experienced neurologist. The training consisted of an explanation of both methods for scoring CDT, followed by the evaluation of a series of CDT pictures obtained from elderly patients with cognitive decline. The training was considered completed when the students were able to score five consecutive CDT pictures adequately according to both methods.

All participants had their drawings photographed, and a code number was attributed to each one for avoiding any breach of confidentiality. The raters independently evaluated all the 404 drawings and scored them according to the Pfizer system and after a week, to the Shulman method. Each rater registered the results in different Excel sheets so that a rater was not aware of the score attributed by the other rater and not even of his own previous (Pfizer) assessment. A third researcher was responsible for data collection into the statistic program, and a fourth one performed the statistical analysis comparing the concordance between the two methods regarding subjects’ cognitive status and the inter-observer correlation.

To assess intra-observer correlation, 52 drawings were randomly reevaluated according to the Pfizer system and the other 52 drawings taking into account the Shulman method, one week apart from the original assessment. Raters were blinded and not aware of their previous scores. As aforementioned, a third researcher collected data and another one performed the statistical analysis.

Quantitative variables were presented as mean and standard deviation or when appropriate, median and 25–75% interquartile range (IQR). This was employed for calculating the inter-observer and intra-observer correlations with kappa statistics (<0.2: poor; 0.21–0.4: weak; 0.41–0.6: moderate; 0.61–0.9: strong; 0.91–1: very strong), through Spearman’s rank correlation coefficient with ordinal scores.^14^ Categorical data were described as a percentage and absolute frequency, including the dichotomized variable normal/abnormal result in both scoring methods, which were compared with the chi-square test. The analyses were performed with commercially available Statistical Package for the Social Sciences (SPSS) version 16.0 (SPSS Inc., Chicago, IL, USA). Statistical significance was assessed with a two-tailed p-value<0.05.

## RESULTS

Demographic and clinical characteristics of the sample are depicted in Table 1. Most of the subjects were Caucasian women.

**Table 1.** Demographic characteristics of the sample (n=404). Qualitative variables are presented as the absolute number and percentage, while quantitative data are exhibited as mean±standard deviation or when otherwise stated, median and interquartile range.

Characteristics	Results		
	Frequency (percentage)	Mean (±SD)	Median (IQR)
Gender			
Female	379 (93.8%)		
Male	25 (6.2%)		
Declared ethnicity			
Caucasian	330 (84.6%)		
Afro-Brazilian Amerindian	52 (13.3%) 8 (2.1%)		
Age (years)		66.9 (7.9)	
Scholarity (years)		10.9 (5.6)	
Pfizer score			3.5 (3.0–4.0)
Shulman score			5.0 (4.0–5.0)

SD: standard deviation; IRQ: interquartile range.

Since the ordinal variables resulted from the scores in the Pfizer and Shulman systems, the nonparametric Spearman’s rank correlation coefficient was employed. There was a strong inter-observer correlation for both methods (Table 2).

**Table 2.** Inter-observer correlation (n=404).

Correlation	r*	95%CI**	Interpretation of correlation
Pfizer test	0.739	0.676–0.796	Strong
Shulman test	0.727	0.667–0.786	Strong

* Spearman’s rank correlation coefficient;

** 95%CI: 95% confidence interval.

A subset of 52 random drawings was randomly reevaluated with the Pfizer system and the other 52 random drawings through the Shulman method. This was performed by both raters, rendering a strong intra-observer correlation, with the exception of the Pfizer system for rater 1, where a moderate correlation was observed (Table 3). There was a strong intra-observer correlation as well when comparing the scores of the Pfizer system with those of the Shulman method, for each rater (n=404; Table 3).

**Table 3.** Intra-observer correlation.

Correlation	r*	95%CI**	Interpretation of correlation
Pfizer			
Rater 1 (n=52)	0.628	0.434–0.776	Moderate
Rater 2 (n=404)	0.702	0.639–0.755	Strong
Shulman			
Rater 1 (n=52)	0.843	0.701–0.946	Strong
Rater 2 (n=52)	0.821	0.704–0.900	Strong
Pfizer vs. Shulman			
Rater 1 (n=404)	0.744	0.681–0.795	Strong
Rater 2 (n=52)	0.819	0.617–0.968	Strong

* Spearman’s rank correlation coefficient with ordinal scores in tests;

** 95%CI: 95% confidence interval.

A weak correlation arose between years of scholarity and the scores in the Pfizer and Shulman methods (Table 4). In contrast, a very weak negative correlation appeared between the scores and age (Table 4).

**Table 4.** Correlation of Pfizer and Shulman scores with years of scholarity and age (n=404).

Correlation	r*	95%CI**	Interpretation of correlation
Pfizer			
Scholarity	0.283	0.189–0.378	Weak
Age	-0.170	-0.267 to −0.073	Very weak
Shulman			
Scholarity	0.244	0.146–0.338	Weak
Age	-0.174	-0.270 to -0.081	Very weak

* Spearman’s rank correlation coefficient with ordinal scores in tests (median of raters);

** 95%CI: 95% confidence interval.

More participants were classified with decreased cognition through the Pfizer method (rater 1: 44.3%; rater 2: 42.1%) than through the Shulman system (rater 1: 26.5%; rater 2: 16.3%) (p≤0.001; n=404).

## DISCUSSION

There are several methods of scoring CDT. Shulman, one of the pioneers of its use, advocates the simpler the scoring system the better because the more complicated and lengthy methods did not appear to add significant value to the psychometric properties or clinical utility of this test.^11,12^ However, even the 5-item score Shulman system was considered to be somewhat difficult.^6^ An easier scoring system based on four items was proposed by Borson et al.^15^ and adopted by the Consortium to Establish a Registry for Alzheimer Disease (CERAD)^16^ and by the Pfizer Inc. and Eisai Inc.,^8^ a fact that popularized the test with the name Pfizer method in some circles.

The main purpose was to compare the Shulman and the Pfizer systems in a large series of adult and elderly people to assess the presence of any difference in terms of inter- and intra-observer ratings. The higher the correlations between the scores, the more significant the results. The inter-observer and intra-observer correlations demonstrated that the Shulman and the Pfizer systems are consistently similar when applied by trained raters. This suggests that the two methods could be applied in primary care, rendering similar results in terms of rating properties. In this setting, the choice of an easier scoring system would be more suitable for cognitive assessment through CDT in primary attention. That is the case with the Pfizer system.

A secondary objective was to compare the interpretation of the results of each method. In the non-selected sample, more participants were classified with decreased cognition with the aid of the Pfizer system than through the Shulman method. Although we did not examine such results in the face of a gold standard diagnostic test, a fact that should be considered a limitation of our study, such finding raises the possibility that the Pfizer method could be more sensitive for screening purposes than the Shulman system. A recent systematic review and meta-analysis about the diagnostic accuracy of CDT proposed its widespread use in the diagnosis of dementia, citing the Shulman method as the most studied, but with some concern about the understanding of its scoring system.^6^ CDT seems to be a robust screening test for Alzheimer’s disease when compared with Mini-Mental State Examination (MMSE).^17^ In contrast, previous studies have already stated that the CDT is not a good screening tool for the diagnosis of mild cognitive impairment.^7,18^ An advisable approach to improve the diagnostic performance of the CDT is the combination with other tests, especially MMSE.^17,19,20^

The scholarity seems to influence the performance in CDT. Small cross-sectional studies suggested that CDT is not sensitive enough to detect very mild dementia neither in educated people^7^ nor in those with limited education.^5^ Illiterate patients can be successfully screened for Alzheimer’s disease using well-known screening instruments in combined protocols (e.g., CDT and MMSE).^21^ A study that analyzed the qualitative elements of CDT, rather than the sole score, showed that among participants without cognitive impairment, those with lower education often presented graphic difficulties, conceptual deficits, and spatial deficits.^10^ Our results confirmed that more educated people exhibit slightly better performance in CDT.

Finally, there is an issue about the age and the performance in CDT. A previous study with 180 adults (47–82 years) found no influence of age in CDT scores.^10^ However, in our sample of 404 adults (50–89 years), a weak negative correlation appeared between the scores and age, that is, the older the subjects, the worse the performance. This finding aligns with a large Brazilian study that also showed a negative correlation between age and cognitive performance, although not employing CDT.^22^

We must recognize the profile of our sample as a limitation since most participants were Caucasian women living in the urban area. This restrains the generalization of our results for other populational groups, namely, men, non-Caucasian, and people living in the countryside. Another concern is that the Elo-Creati cohort is representative of adult and elderly people engaged in occupational activities related to healthy aging, far from a sample of patients with declared cognitive decline or dementia. Thus, our population seems of a primary care subset, instead of secondary or tertiary care. Our conclusions may not apply to groups composed solely of patients with cognitive problems.

In summary, our results suggest that the simplest form of scoring CDT called the Pfizer system can be employed in primary care for evaluating cognitive decline, with an easier interpretation than the Shulman method. The combination of CDT with other cognitive tests, especially MMSE, is advisable for a thorough evaluation, taking into account educational level and age as influencing factors.
